# Fibromodulin Gene Variants (*FMOD*) as Potential Biomarkers for Prostate Cancer and Benign Prostatic Hyperplasia

**DOI:** 10.1155/2022/5215247

**Published:** 2022-05-31

**Authors:** Tamara Silva, Caroliny Pinto Gomes, Danielle Dutra Voigt, Ritiele Bastos de Souza, Karoline Medeiros, Nicole Lima Cosentino, Ana Carolina Proença Fonseca, Tatiana Martins Tilli, Enrique Antonio Covarrubias Loayza, Vivianne Galante Ramos, Pedro Hernán Cabello Acero

**Affiliations:** ^1^National Institute of Metrology, Quality and Technology, Rio de Janeiro, Brazil; ^2^Human Genetics Laboratory, Oswaldo Cruz Institute, Oswaldo Cruz Foundation, Rio de Janeiro, Brazil; ^3^Laboratory of Genetics, School of Health Science, University of Grande Rio/AFYA, Rio de Janeiro, Brazil; ^4^Laboratory of Immunopharmacology, Oswaldo Cruz Institute, Oswaldo Cruz Foundation, Rio de Janeiro, Brazil; ^5^Translational Oncology Platform, Center for Technological Development in Health, Oswaldo Cruz Foundation, Rio de Janeiro, Brazil; ^6^Laboratory of Cardiovascular Investigation, Oswaldo Cruz Institute, Oswaldo Cruz Foundation, Rio de Janeiro, Brazil; ^7^Department of Health-Duque de Caxias Polyclinic, School of Health Science, University of Grande Rio/AFYA, Rio de Janeiro, Brazil

## Abstract

By the year 2050, the world's elderly population may increase exponentially, raising the rate of disease characteristic of this group, such as prostate cancer (PCa) and benign prostatic hyperplasia (BPH). Prostate disorders have a multifactorial etiology, especially age and genetic factors. Currently, PCa is the second most frequent neoplasm in the male population worldwide. The fibromodulin gene encodes a small leucine-rich proteoglycan (SLRP) which acts in the collagen fibrillogenesis pathway, cell adhesion, and modulation of TGF-*β* signaling pathways, which has been recently associated with PCa. The present study sequenced the coding region of the *FMOD* in a sample of 44 PCa, 90 BPH, and 82 controls from a Brazilian population, and the results identified 6 variants: 2 missenses (p.(Tyr42Ser) and p.(Pro24Ala)); 3 synonymous (p.(His253=), p.(Asn353=), and p.(Glu79=)); and 1 intronic (c.980-114A>G). Of these, p.(Tyr42Ser), p.(Pro24Ala), and p.(Asn353=) are rare variants, and p.(Tyr42Ser) was predicted as potential pathogenic by the algorithms used here, in addition to not being observed in controls, suggesting that may be a potential biomarker for development of PCa and BPH. In conclusion, we identified for the first time, in Brazilian individuals with PCa and BPH, a potentially pathogenic variant in the analysis of *FMOD* gene. Further studies are needed to investigate the deleterious effect of this variant on the structure and/or function of the FMOD protein.

## 1. Introduction

Prostate cancer (PCa) is the most common noncutaneous cancer in men worldwide, with an estimated 1,600,000 cases and 366,000 deaths per year [1]. PCa is the second most frequent tumor in American men, estimating 248,530 new cases and 34,130 deaths in 2021 [2]. According to Brazilian National Cancer Institute (INCA), PCa is the second most common cancer among Brazilian men and, for 2021, more than 65,840 new cases were expected [3].

Screening for detection and monitoring of PCa is one of the most controversial question in the field of urology. This is because the primary tests commonly used in the early detection of neoplasia are the digital rectal examination (DRE) and the PSA test (prostate specific antigen). Additionally, other tests are necessary to confirm the diagnosis, as the transrectal ultrasound (TRUS) and biopsy [4, 5].

Although the association between the density of prostate-specific antigen (PSA), benign prostatic hyperplasia (BPH), and PCa is consolidated in the literature [6, 7], several studies involving potentially more sensitive and specific biomarkers have been developed with the aim of improving the detection and monitoring of these pathologies and eliminating the limitations inherent to PSA [8, 9].

The PSA is not specific for prostate cancer; many benign conditions can elevate PSA levels, such as simple urinary tract inflammation or even BPH, leading to false-positive results [10, 11]. Some studies have also revealed that some individuals with PCa may have PSA levels below the reference threshold (4.0 ng/mL), thus leading to false-negative results, demonstrating that there are still no reliable values in PCa investigation [12]. While there is no evidence that the inflammatory process or BPH leads to PCa, it is possible for some individuals to develop such conditions and also prostate cancer [13].

Cancer is characterized by abnormal cell proliferation due to loss of control of cell division and the apoptosis process, leading to tumor formation [14, 15]. Any cell type needs to inhabit the extracellular matrix (ECM) and interact with its components that can play important roles in tumorigenesis [16]. Several small leucine-rich proteoglycans (SLRP) that are part of ECM, as class I biglycan, decorin, fibromodulin (FMOD), and class II lumican, favor the collagen fibrillogenesis regulation [17]. Among these, the proteoglycan FMOD has emerged with interesting particularities that involve several crucial biological processes, as angiogenesis, migration, and apoptosis [16].

Some studies have been indicated that fibromodulin may modulate certain signaling pathways, such as vascular endothelial growth factor (VEGF), fibroblast growth factor-2 (FGF-2), and especially, transforming growth beta (TGF-*β*) [18]. Moreover, fibromodulin also acts in the initiation and progression of pathologies like B-cell chronic lymphocytic leukemia [19].

FMOD function goes far beyond the regulation of collagen fibrillogenesis and cell adhesion. Fibromodulin is able to inhibit VEGF expression, suggesting that proteoglycan regulates other signaling pathways besides TGF-*β* [16, 20]. FMOD is also considered an efficient and fundamental angiogenic factor in lung cancer, in wound healing process and optical as well as skin diseases [18, 21, 22]. Therefore, FMOD functions are mainly related to angiogenesis process, suggesting as a potential therapeutic target to cancer and also other conditions associated with abnormal angiogenesis [21, 23].

Due to its product be detected in body fluids as urine, blood, or prostatic secretions, the fibromodulin coding gene has great potential to be used as a new biomarker to diagnosis of patients with benign or malignant prostate tumors [19, 24]. Therefore, to understand FMOD influence in several physiological processes to development of PCa is very necessary. Up to our knowledge, screening of FMOD gene is the first study in a sample with PCa and BPH in Brazil. Thus, we hope that the results of this study will contribute to the improvement of the diagnosis of these prostatic disorders.

## 2. Material and Methods

### 2.1. Study Population

This cross-sectional observational study included 216 unrelated male patients, aged plus 55 years from Rio de Janeiro State, Brazil. All were examined by urologists and submitted to DRE, PSA, and biopsy tests. Individuals considered healthy for this study (mean age of 60.82 ± 9.41) had a PSA level < 4 ng/mL, an unaltered rectal examination, and no other history of neoplasia. The diagnosis of PCa was made based on the altered DRE, the PSA quantification (>4 ng/mL), and the positive biopsy for malignancy. The diagnosis of BPH was made based on the altered clinical characteristics, the PSA quantification (>4 ng/mL), and the altered prostate volume.

Our cohort was divided into 44 with PCa, 90 with BPH, and 82 healthy controls. The Research Ethics Committee of the National Cancer Institute José Alencar Gomes da Silva (CAE: 88510618.8.30025274) and the University of Grande Rio (CAE: 8810618.8.0005253) approved the research protocol and the free and informed consent term, obtained from all participants.

### 2.2. Molecular Analyses

Genomic DNA was isolated from peripheral blood leukocytes using the QIAamp DNA Blood (Qiagen, Hilden, Germany). To assess the presence of variants in the *FMOD* gene, we amplified two exons and intron-exon boundaries by the polymerase chain reaction (PCR) technique using 6 pairs of oligonucleotides (IDT-Integrated DNA Technologies) (Supplementary Material Table S1), followed by Sanger sequencing. The conditions used in the PCR are shown in Supplementary Material Table S2.

The PCR products were purificated using the enzyme ExoSAP-IT (Applied Biosystems, Vilnius, Lithuania). Then, these products were sequenced using the Big Dye Terminator v3.1 kit (Applied Biosystems, Austin, TX, USA) and processed on an ABI 3130 Genetic Analyzer (Applied Biosystems Inc. US). The sequences were aligned with the reference sequence (ENST00000354955.4) and were analyzed by BioEdit Sequence Alignment Editor software v7.2.6.1 (Isis Pharmaceuticals).

### 2.3. Bioinformatic Tools

The identified *FMOD* variants were investigated in order to ascertain their previous occurrence in public databases, such as the Online Archive of Brazilian Mutations (http://abraom.ib.usp.br/index.php), project database 1000 Genomes (http://www.internationalgenome.org), Clinvar (http://www.ncbi.nlm.nih.gov/clinvar/), dbSNP (https://www.ncbi.nlm.nih.gov/), ExAC Browser (http://exac.broadinstitute.org), Human Genome Mutation Database (HGMD) (https://pubmed.ncbi.nlm.nih.gov/), and PubMed (https://pubmed.ncbi.nlm.nih.gov/).

To assess the potential functional impact of the identified missense variants, we used in silico prediction tools including the SIFT4G [25], PolyPhen-2 [26], PROVEAN [27], WS-SNPs & GO [28], MutPred [29], SNAP [30], Fathmm [31], M-PCA [32], mutation assessor [33], PANTHER-PSEP [34], mutation taster [35], and Revel [36].

### 2.4. Statistics Analysis

We used IBM® SPSS software (V.22.0) for descriptive and inferential analyses. In the case of normally distributed variables, comparisons were made between groups using the Student's *t* and ANOVA tests (for variables: age, body mass index, and prostate weight); subsequently, Student-Newman-Keuls (SNK) post hoc analyses were performed to complement ANOVA. In relation to PSA_T_ scores, we had observe its nonnormal distribution, being influenced by extremely high values observed in three PCa patients, so the comparisons among groups were made using Kruskal-Wallis nonparametric test. All these inferences were made at the 5% level of significance.

## 3. Results

### 3.1. Basic Clinical Characteristic

This study comprised 216 individuals, stratified into 44 PCa patients, 90 with BPH, and 82 healthy controls, whose clinical characteristics are showing in Table 1. As expected, we observed that PSA_t_, PSA_f_, and prostate weight were statistically different among groups.

Table 1 presents the results of the comparisons made between the three sample groups.

### 3.2. *FMOD* Molecular Screening

**Figure 1:**
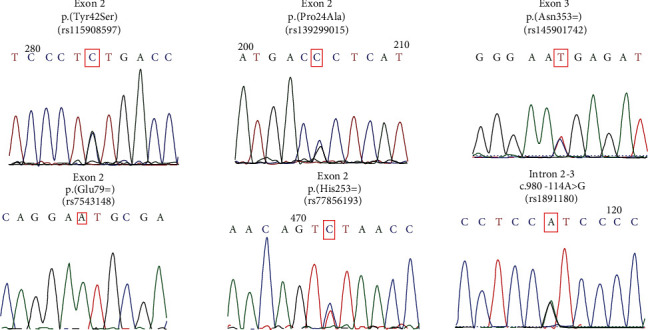
Electropherograms referring to alterations found in the FMOD gene tracking.

In this study, we sequenced the coding region of the *FMOD* gene in 134 probands and 82 controls from a sample from Rio de Janeiro. Our results showed 6 variants, in which 2 were missenses [(p. (Tyr42Ser) and p. (Pro24Ala)], 3 synonymous (p. His253=, p. Asn353=, and p. Glu79=), and 1 intronic (c.980-114A>G) (Table 2). The electropherograms are presented in Figure 1.

The missense variant p.(Tyr42Ser) was present in 2 hyperplastic patients and 1 neoplastic patient. This variant was absent in the control group. The p.(Pro24Ala) variant was observed in heterozygosity in a control patient. Regarding the synonymous variants, p.Asn353= was found in heterozygosity in 1 neoplastic proband, and p.His253= was found in two neoplastic patients and one control in heterozygosity. The p.Glu79= was common in our sample, which was found in 44 neoplastic probands, 70 hyperplastic, and 66 controls. This variant was observed in heterozygosity and homozygosity state. Additionally, the intronic variation c.980-114A>G was observed in 44 neoplastic, 74 hyperplastic, and 82 healthy individuals. Therefore, both p.Glu79= and c.980-114A>G variants were characterized as polymorphism.

**Figure 2:**
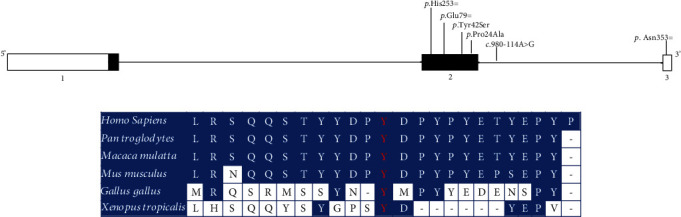
Schematic representation of the FMOD gene and protein domains. (a) Alterations found are pointed out in the gene scheme. (b) Mutation taster alignment of the FMOD between species.

The p.(Tyr42Ser) is presented in exon 2 and is characterized by the exchange of the amino acid tyrosine for serine at position 42 of the protein. In our sample, we identified this change in heterozygosity in 3 individuals, of which two were BPH group aged 72 and 74 years and in one individual was the PCa group aged 75 years. The tyrosine residue at position 42 of fibromodulin is evolutionarily conserved across species (Figure 2). The SIFT4G Prediction, PolyPhen-2_HVAR, MutPred2, SNAP, PANTHER-PSEP, and mutation taster tools (Table 3) predicted this variant to be probably harmful. Tyrosine and serine are polar amino acids that have no charge, but tyrosine contains a phenol group attached to its side chain while serine has a hydroxylated methyl group. On the other hand, the p.(Pro24Ala) located in exon 2 of the *FMOD* gene results in the exchange of the amino acid proline to alanine at position 24 of the protein. This variant, found in a 57-year-old control individual, was predicted as benign by almost all silico tools used (Table 3).

## 4. Discussion

In recent years, changes in mortality and age pyramid structure of the global population in recent years has resulted in a substantial increase in complex diseases [37]. This scenario brings a progression of numerous diseases, including cancer, which is one of the main causes of death in several countries [38, 39]. With respect to PCa, the incidence rates by age increase sharply from the age of 45 years [40]. Despite the numerous efforts of the world medical community to raise awareness of prostate cancer, there is still great resistance from the male population about the care of their health.

Mutations or polymorphisms in many genes are described in the literature as influencing the development of PCa and possibly BPH [41–48]. The *FMOD* gene, which encodes fibromodulin, plays an important role in the regulation of collagen fibrillogenesis, angiogenesis, reprogramming of human fibroblasts into pluripotent cells, and modulation of TGF-*β* activity and is associated with metastatic phenotypes. Interestingly, until recently, this gene had not been associated with prostatic pathologies [19, 24].

Bettin and Reyes (2016) showed the strong interaction between prostate cancer and *FMOD*, revealing that the gene is highly expressed in human PCa cells, whereas in hyperplastic cells, there is no increase expression. In our research, *FMOD* gene was sequenced in 216 individuals. A total of six alterations were observed p.(Tyr42Ser), p.(Pro24Ala), p.His253=, p.Asn353=, and c.980-114A>G, of which p.(Tyr42Ser) was rare and predicted to be pathogenic by our in silico analyses. It is important to emphasize that none of these alterations were associated with PCa or BPH and neither with any other pathology, prior to our study.

The missense p.(Tyr42Ser) alteration was identified in individuals with prostatic alterations, presenting a frequency in our population of 1.6%. In contrast, the p.(Pro24Ala) was found in 1 individual of the control group. One of the synonymous variants found was p.His253=, located in exon 2 of the *FMOD* gene and characterized by the exchange of a cytosine for a thymine (c.759C>T). This change was observed in the study sample in three individuals, with two of them allocated in the PCa group (57 and 74 years) and one in the control group (65 years) representing 1,4% of its frequency in our study.

Another synonymous variant p.Asn353= (c.1059C>T) found in exon 3 was observed in heterozygosis in an individual with PCa aged 64 years, being absent in the healthy individuals analyzed. This synonymous alteration was identified in individuals with prostatic alterations, presenting a frequency in our population of 0.49%. However, even though the p.His253= and p.Asn353= variants do not present amino acid alterations according to the Human Splicing Finder tool, the base exchange may be located in the splicing site, so we cannot rule out its potential modifying effect on the protein. In this context, further functional studies are necessary to elucidate it.

The other two alterations, p.Glu79= and c.980-114A>G, showed a polymorphic behavior, being very frequent in our sample. The frequency of the p.Glu79= polymorphism in our study was 70.67%, whereas the frequency of the c.980-114A>G was 61.54%. However, after performing genotypic and allelic association analyzes, no significant associations were found for any of the groups analyzed (*p* > 0.05) in relation to these variants. It is to our knowledge that the p.Glu79= variant was previously studied in relation to other pathologies, as myopia and the risk of tearing the anterior cruciate ligament; however, it has not been associated with such diseases [49, 50].

In conclusion, we identified a potentially pathogenic variant in *FMOD* gene in a cohort with Brazilian individuals with PCa and BPH. As far as we know, this is the first study to screen part of the *FMOD* gene, analyzing its association with prostate cancer and benign prostatic hyperplasia. We believe that the functional studies are needed to better understand the role of the alterations found here, especially the missense ones, in the mentioned pathologies and their contribution to the clinical profile of patients.

## Figures and Tables

**Table 1:** Comparative analysis of quantitative variables between sample groups.

Variables	Control		BPH		PCa		*p*	*p*1	*p*2	*p*3
	*N*	Values	*N*	Values	*N*	Values				
Age (years)	82	60.82 ± 9.41	90	69.80 ± 8.12	44	71.27 ± 7.64	<0.01	<0.05	<0.05	>0.05
BMI (kg)	75	26.12 ± 3.35	84	26.26 ± 3.93	24	27.35 ± 3.83	0.509	>0.05	>0.05	>0.05
PSA_T_ (ng/mL)	82	0.00 ± 0.87	90	2.54 ± 3.76	44	11.52 ± 16.95	<0.01	—	—	—
PSA_F_ (ng/mL)	82	0.00 ± 0.23	90	0.43 ± 0.69	44	0.00 ± 1.29	<0.01	—	—	—
Prostate weight (g)	21	30.15 ± 9.87	52	49.28 ± 21.54	20	46.30 ± 26.30	0.947	<0.05	<0.05	>0.05

Note: values indicate median ± interquartile range; *p* is the probabilistic value used to measure the significance of differences between the means of variables. *p*1 is the probabilistic value used to measure the significance of differences between the control and BPH groups. *p*2 is the probabilistic value used to measure the significance of differences between the control and PCa groups. *p*3 is the probabilistic value used to measure the significance of differences between the BPH and PCa groups. Some variables were not passed on in part of the studied group. PSA_T_: total prostate specific antigen; PSA_F_: free prostate specific antigen; SBP: systolic blood pressure; DBP: diastolic blood pressure; BMI: body mass index; source: elaborated by the author.

**Table 2:** Variants found in the molecular analysis of the *FMOD* gene.

Gene location	SNP ID	c.DNA	Protein	Type of change	MAF	No. of individuals with alteration		
						Control	BPH	PCa
Exon 2	rs115908597	c.125A>C	p.(Tyr42Ser)	Missense	<0.01	0	2	1
Exon 2	rs139299015	c.70C>G	p.(Pro24Ala)	Missense	<0.01	1	0	0
Intron 2-3	rs1891180	c.980-114A>G	—	Intronic	0.60	82	74	44
Exon 2	rs77856193	c.759C>T	p.(His253=)	Synonym	<0.01	1	0	2
Exon 2	rs7543148	c.237G>A	p.(Glu79=)	Synonym	0.40	66	70	44
Exon 3	rs145901742	c.1059C>T	p.(Asn353=)	Synonym	0.04	0	0	1

Representation of the variants found through the automatic sequencing of the *FMOD* gene, with its nitrogenous base and/or amino-acid base exchange characteristics, in addition to the number of individuals found with the alteration in each of the sample groups. Source: prepared by the author.

**Table 3:** In silico prediction of missense changes identified in the *FMOD* gene.

Prediction tool	p.(Tyr42Ser) Y42S		p.(Pro24Ala) P24A		Results	Website	Ref.
	Score	Prediction	Score	Prediction			
SIFT4G predictions	0.004	Deleterious	0.339	Tolerated	Score range: 0 to 1 (≤0.05 damaging/>0.05 tolerated).	https://sift.bii.a-star.edu.sg/www/SIFTdbSNP.html	[25]
PolyPhen-2_HVAR	0.831	Probably damaging	<0.01	Benign	Score range: 0 (benign) to 1 (damaging). Probably damaging, possibly damaging or benign.	http://genetics.bwh.harvard.edu/pph2/index.shtml	[26]
PROVEAN	-0.86	Neutral	-0.56	Neutral	Default score threshold: -2.5 (≤-2.5 deleterious/>-2.5 neutral).	http://provean.jcvi.org/	[27]
WS-SNPs & GO	0.280	Neutral	0.106	Neutral	Score range: 0 to 1 (probability score: >0.5 disease-associated).	http://snps.biofold.org/snps-and-go/	[28]
MutPred2	0.625	Possibly pathogenic	0.209	Neutral	Score range: 0 to 1 (general pathogenicity score: ≥0.50).	http://mutpred.mutdb.org/#qform	[29]
SNAP	2	Effect	-54	Neutral	Score: -100 to 100 (≥1 effect).	http://www.rostlab.org/services/SNAP	[30]
FATHMM	0.52	Tolerated	0.67	Tolerated	Pathogenicity threshold: <0 (dano). >0 (Tolerado).	http://fathmm.biocompute.org.uk/inherited.html	[31]
M-CAP	∗	∗	0.003	Benign	Pathogenicity threshold: >0.025.	http://bejerano.stanford.edu/mcap/	[32]
Mutation assessor	1.04	Low impact	0.345	Neutral	Score cutoff: 0.8 neutral and low impact/1.9 low impact and medium impact/3.5 medium impact and high impact.	http://mutationassessor.org/r3/	[33]
PANTHER-PSEP	455	Possibly pathogenic	91	Possibly benign	Length of time: >450 my probably damaging/450 my > time > 200 my possibly damaging/<200 my probably benign.	http://www.pantherdb.org/tools/csnpScore.do	[34]
Mutation taster	0.999999999606647-A	Disease causing	0.999999999606647-P	Polymorphism	Prediction: A. Disease causing: probably deleterious/D. disease causing automatic: deleterious/N. polymorphism: probably harmless/P. polymorphism automatic: harmless.	http://www.mutationtaster.org/	[35]
Revel	0.168	Benign	0.081	Benign	Score range: 0 to 1 (>0.50 likely disease causing/<0.50 likely benign).	https://sites.google.com/site/revelgenomics/downloads	[36]

Notes: ∗MCAP-MCAP scoring is not available for some alleles; location 1 : 203317274. Abbreviation: my: millions of years.

## Data Availability

The present study deals with the tracking of the FMOD gene, in Brazilian individuals with PCa and BPH that showed new allelic variants, among which one potentially pathogenic. We believe that this information can significantly contribute to expanding our biological knowledge, prevention, early diagnosis, and treatment of prostate cancer and benign prostatic hyperplasia.

## Supplementary Materials

Supplementary Materials All information presented in this study are products of analysis of polymorphisms identified by PCR and Sanger sequencing, which are available in the article and in the supplementary material (S1, S2, and S3).
