# Supplementary material for: Fibromodulin Gene Variants (FMOD) as Potential Biomarkers for Prostate Cancer and Benign Prostatic Hyperplasia
Source: Dis Markers. 2022 May 31;2022:5215247. doi: 10.1155/2022/5215247 (PMC9173908; doi:10.1155/2022/5215247)
Supplement: Supplementary Materials — All information presented in this study are products of analysis of polymorphisms identified by PCR and Sanger sequencing, which are available in the article and in the supplementary material (S1, S2, and S3). [file 5215247.f1.zip › 5215247.f1/Supplementary material S1 (1).docx]

**Fibromodulin gene variants *(FMOD)* as potential biomarkers for Prostate Cancer and Benign Prostatic Hyperplasia**

**Supplementary material**

DNA sequencing

To evaluate the presence of variants in the *FMOD* gene, we amplified the DNA of the 216 probands for two exons and intron-exon boundaries using the polymerase chain reaction (PCR) technique using 3 pairs of oligonucleotides (IDT-Integrated DNA Technologies), described in Table S1, followed by Sanger sequencing. Genomic DNA was isolated from peripheral blood using standard procedures. The Table S2 shows the conditions used in the PCR and the Table S3, the denaturation, annealing and extension temperatures, for amplification of the fragments corresponding to the 2 exons of the *FMOD* gene. The PCR products were subjected to purification using the enzyme ExoSAP-IT (Thermo Scientific Inc. US), following the protocol described by the manufacturer. To verify the presence of mutations, bidirectional sequencing using the Big Dye Terminator v3.1 Kit (Life Technologies Inc. US) was conducted on an ABI 3130 Genetic Analyzer automatic sequencer (Applied Biosystems Inc. US) and the sequences were analyzed by the software BioEdit Sequence Alignment Editor version v7.2.6.1 (Isis Pharmaceuticals) and the sequences obtained were aligned with the fragment corresponding to the wild sequence of the *FMOD* gene (Transcript: ENST00000354955.4), accessing the online database ensemble.

# Table S1. Design of *FMOD* gene primers.

| Oligo | Primer sequences (5'-3') | Base pair | T_m °C_ |
| --- | --- | --- | --- |
| FMOD 2.1 | F: 5’-ccgaagaaatgagagcaagg-3’  R: 5’-TACAAGGCCGTGAGGTTCTC-3’ | 777pb | 61ºC |
| FMOD 2.2 | F: 5’-GTGATAAGGTGGGCAGGAAG-3’  R: 5’-tcaggttgcttcatttgtgc-3’ | 681pb | 62ºC |
| FMOD 3.0 | F: 5’-ttggtggttaggaggtgagc-3’  R: 5’-CGTGGACTTCTGTCACATGG-3’ | 489pb | 62ªC |

Note: Tm: Melting temperature (annealing); exon 2 of the gene was divided into exon 2.1 and 2.2 because of its extensive size; F=Foward 5’→ 3’ e R=Reverse 3’→5’.

Table S2. Conditions used in PCR to amplify fragments corresponding to exons of the *FMOD* gene

| **Reagents** | **Measure** | |
| --- | --- | --- |
| Reaction Buffer (10X) | 1X |  |
| MgCl2 (50 mM) | 1 mM |  |
| dNTP (5mM) | 200 μM |  |
| Oligonucleotide F (10mM) | 1 mM |  |
| Oligonucleotide R (10mM) | 1 mM |  |
| Platinum™Taq DNA Polimerase (1U/μL) | 1 U |  |
| ADN (50 ηg/μL) | 1 μL |  |
| Final Volume | 25μL |  |

*10X Buffer, Mg free (Tris-HCl 200 mM, pH 8,4, KCl 500 mM).

Table S3. Cycling conditions used in the PCR for the mutational screening of the *FMOD* gene

| **Phases** | **Exons 2 and 3** |
| --- | --- |
| Initial denaturation | 94°C – 3’ |
| Denaturation | 94°C – 45’’ |
| Annealing | 62°C – 30’’ 40x |
| Extension | 72°C – 1’30 |
| Final extension | 72°C – 10’ |

x = number of cycles
