# Supplementary material for: Fibromodulin Gene Variants (FMOD) as Potential Biomarkers for Prostate Cancer and Benign Prostatic Hyperplasia
Source: Dis Markers. 2022 May 31;2022:5215247. doi: 10.1155/2022/5215247 (PMC9173908; doi:10.1155/2022/5215247)
Supplement: Supplementary Materials — All information presented in this study are products of analysis of polymorphisms identified by PCR and Sanger sequencing, which are available in the article and in the supplementary material (S1, S2, and S3). [file 5215247.f1.zip › 5215247.f1/Supplementary material S2.pdf]

Exon 2
p.(Tyr42Ser)
(rs115908597)

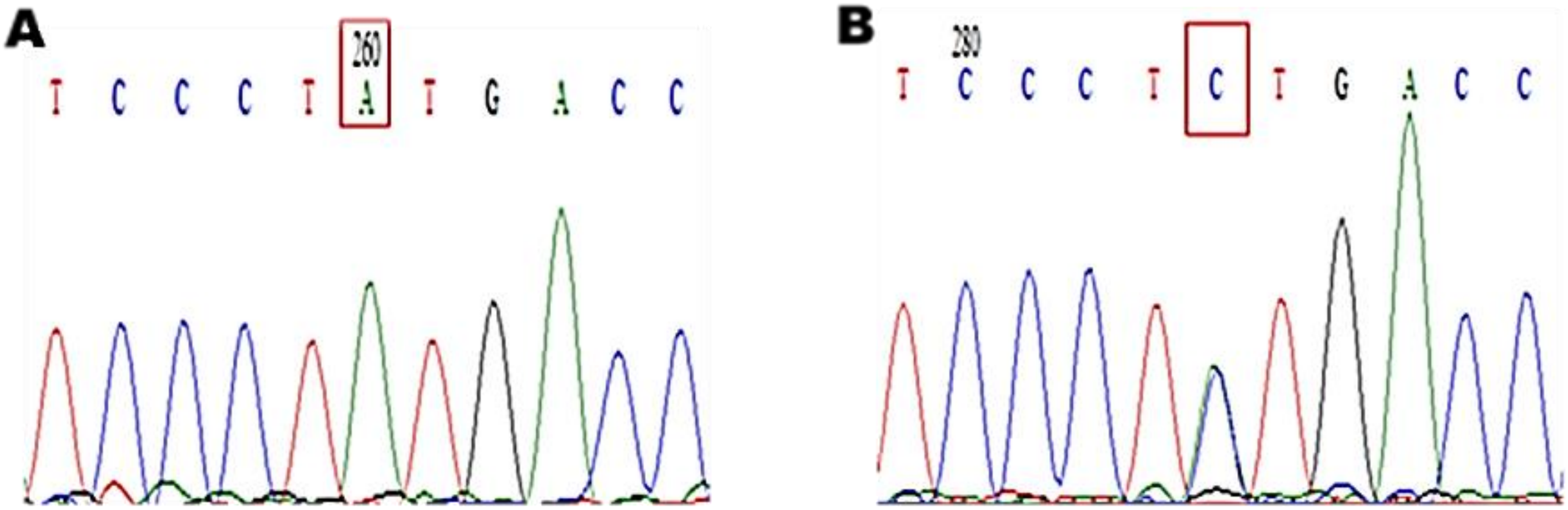

A) Wild-type sequence of the *FMOD* gene. B) Alteration c.125A>C (rs115908597) in heterozygosity in the *FMOD* gene. Source: elaborated by the author.

Exon 2
p.(Pro24Ala)
(rs139299015)

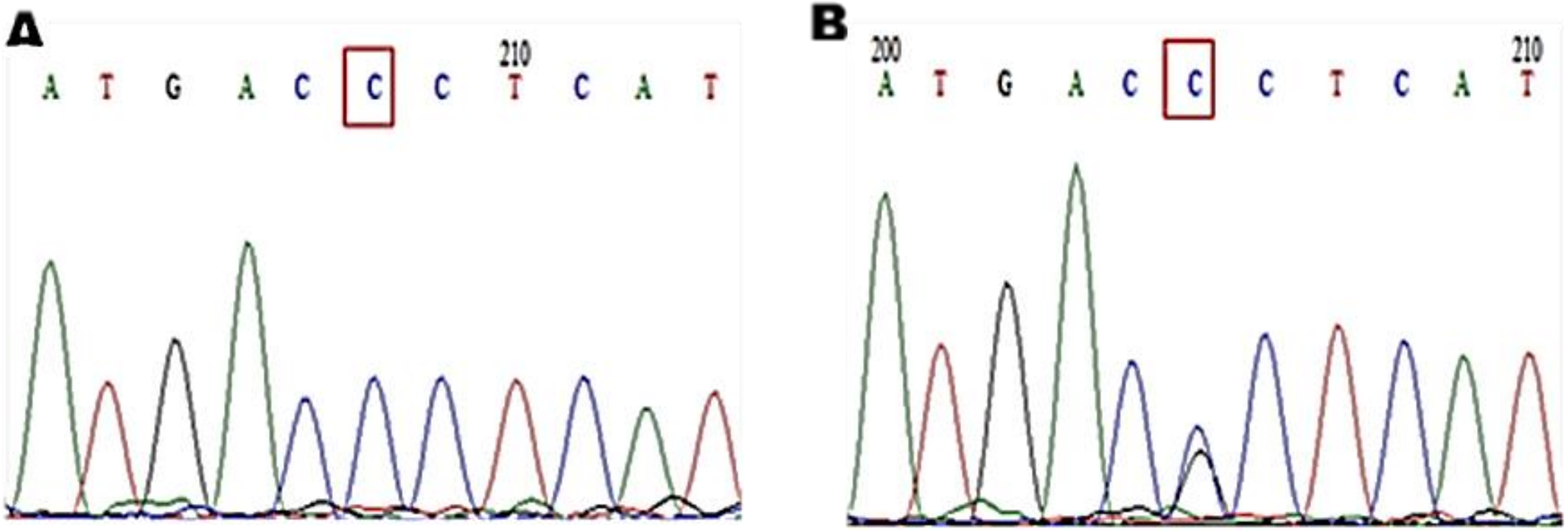

A) Wild-type sequence of the *FMOD* gene. B) Alteration c.70C>G (rs139299015) in heterozygosity in the *FMOD* gene.
Source: elaborated by the author.

Exon 3
p. (Asn353=)
(rs145901742)

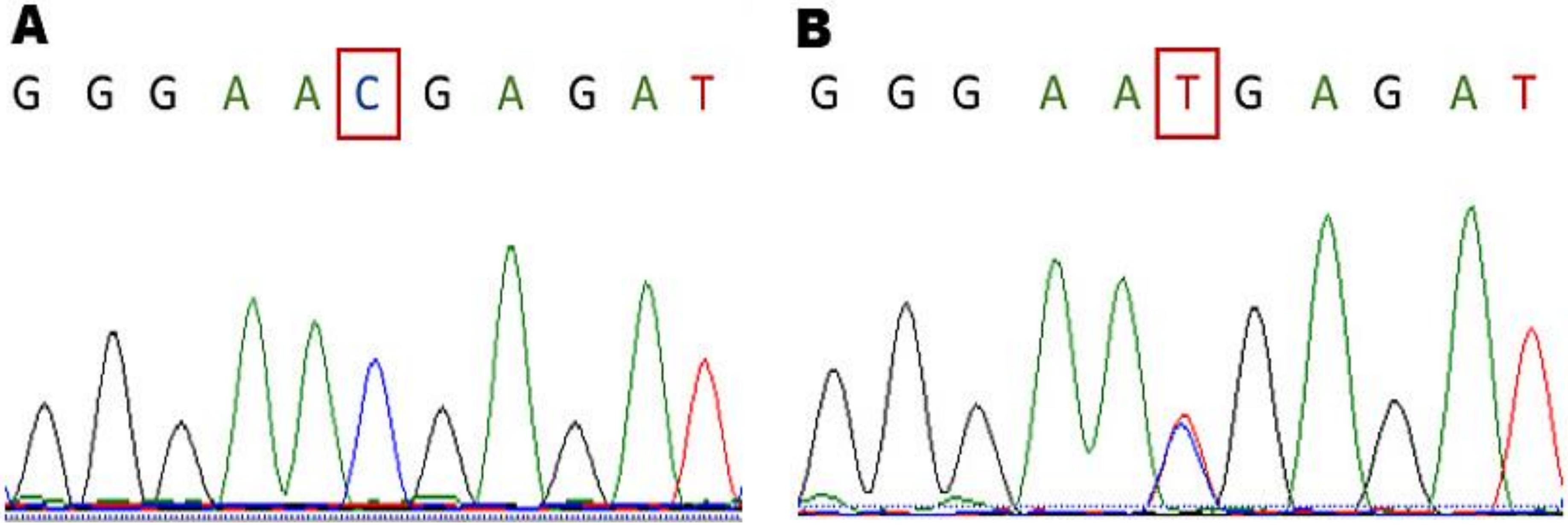

A) Wild-type sequence of the *FMOD* gene. B) Alteration c.1059C>T (rs145901742) in heterozygosity in the *FMOD* gene. Source: elaborated by the author.

Exon 2
p.(Glu79=)
(rs7543148)

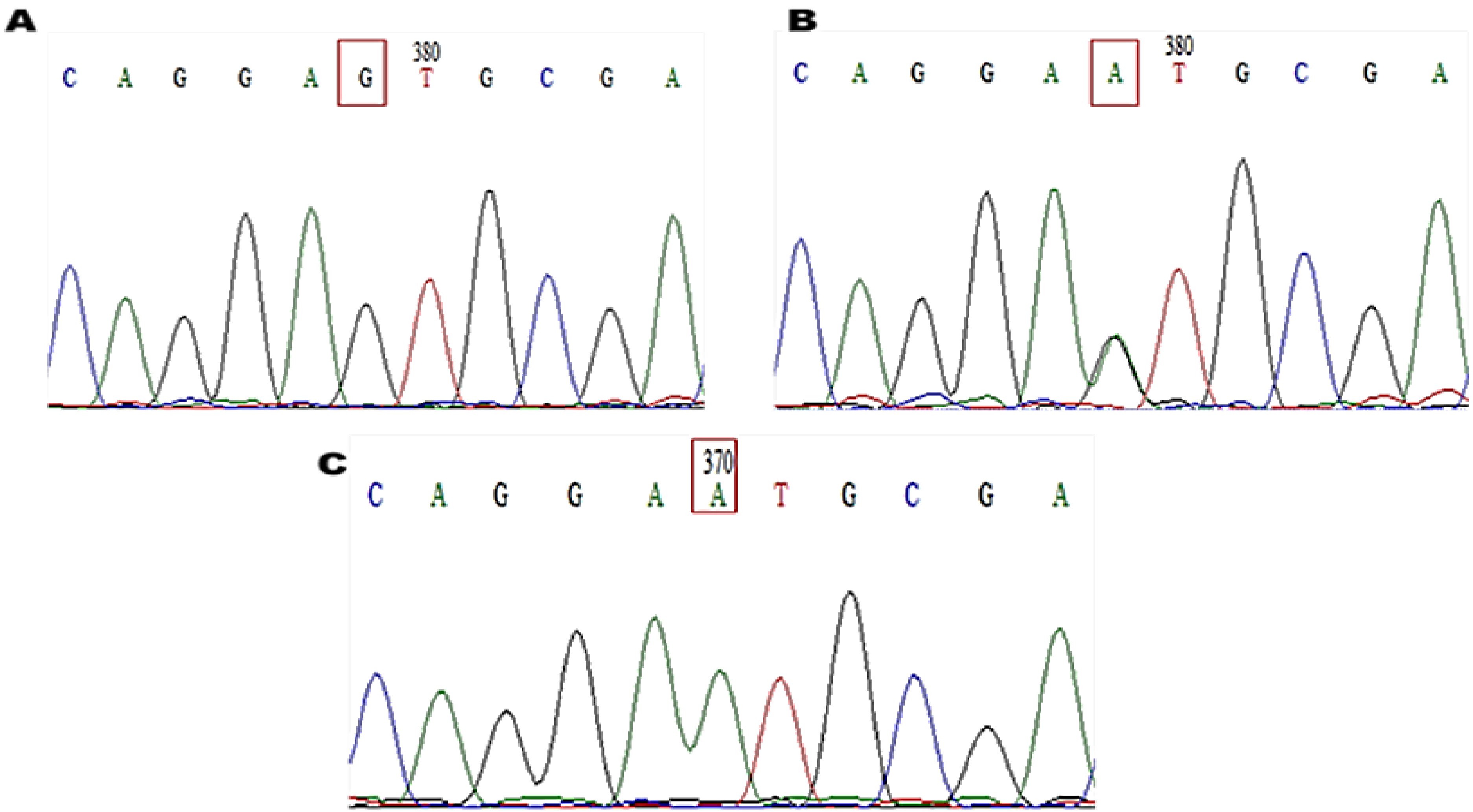

A) Wild-type sequence of the *FMOD* gene. B) Alteration c.237G>A (rs7543148) in heterozygosity in the *FMOD* gene. C) Alteration c.237G>A (rs7543148) in homozygosity in the *FMOD* gene. Source: elaborated by the author.

Exon 2

p.(His253=)

(rs77856193)

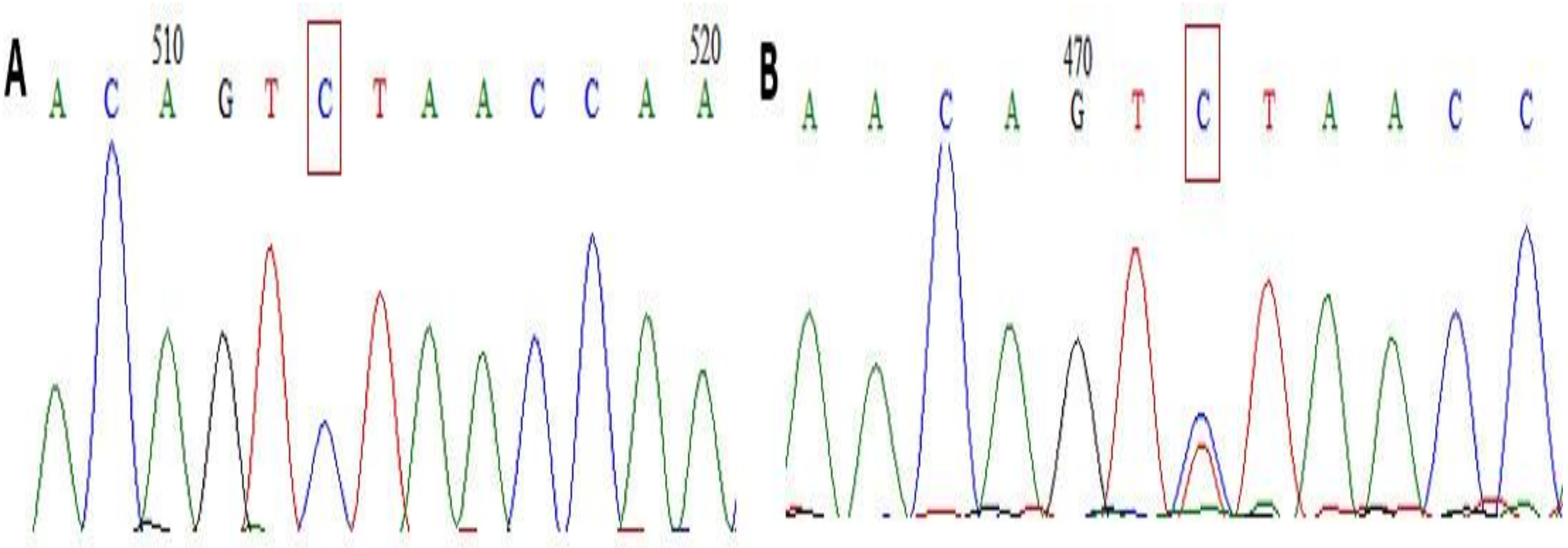

A) Wild-type sequence of the *FMOD* gene. B) Alteration c.759C>T (rs77856193) in heterozygosity in the *FMOD* gene. Source: elaborated by the author.

Intron 2-3

c.980-114A>G

(rs1891180)

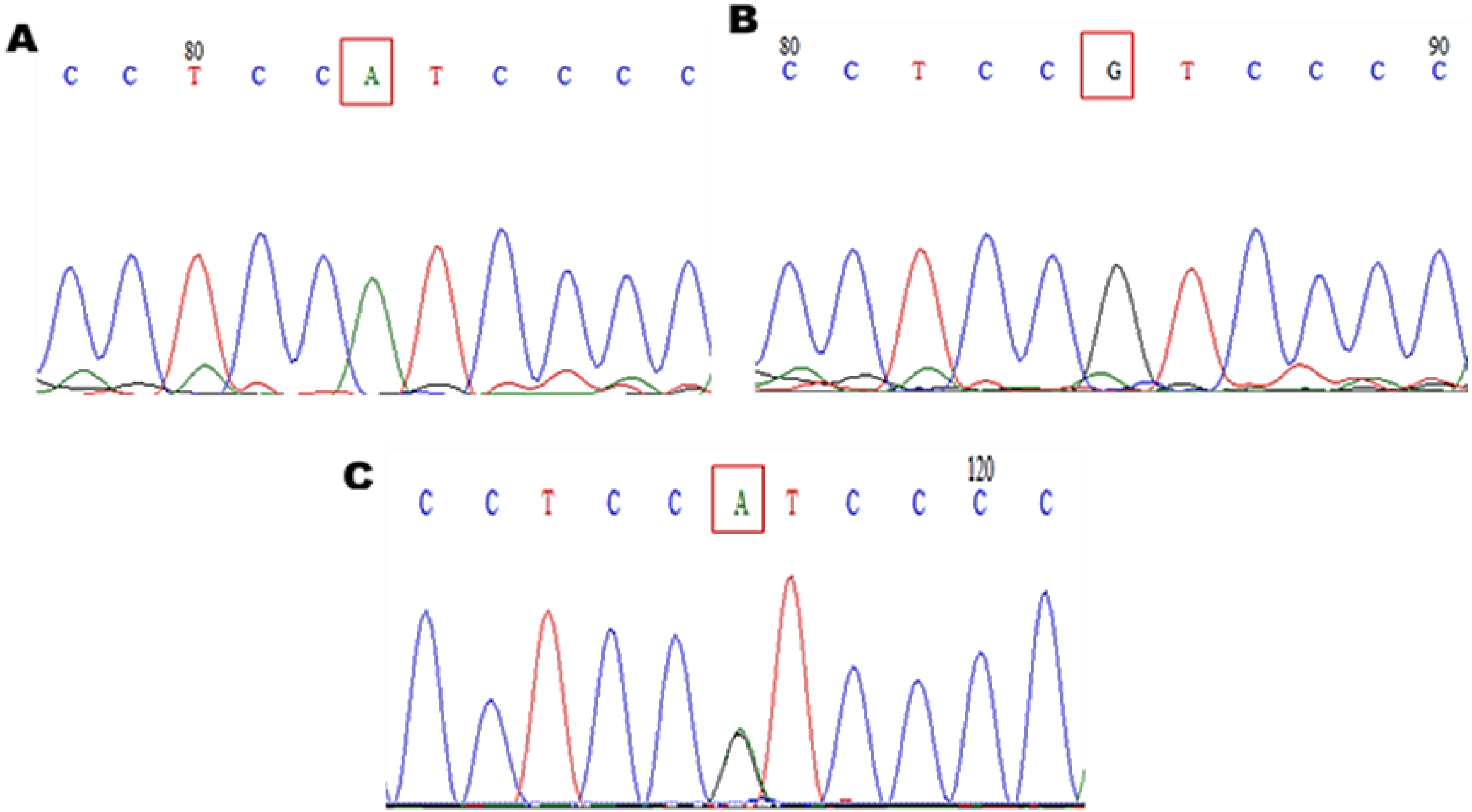

A) Wild-type sequence of the *FMOD* gene. B) Alteration c.980-114A>G (rs1891180) in homozygosity in the *FMOD* gene. C) Alteration c.980-114A>G (rs1891180) in heterozygosity in the *FMOD* gene. Source: elaborated by the author.
