# Supplementary material for: Fibromodulin Gene Variants (FMOD) as Potential Biomarkers for Prostate Cancer and Benign Prostatic Hyperplasia
Source: Dis Markers. 2022 May 31;2022:5215247. doi: 10.1155/2022/5215247 (PMC9173908; doi:10.1155/2022/5215247)
Supplement: Supplementary Materials — All information presented in this study are products of analysis of polymorphisms identified by PCR and Sanger sequencing, which are available in the article and in the supplementary material (S1, S2, and S3). [file 5215247.f1.zip › 5215247.f1/Supplementary material S3.pdf]

Prediction tool	Tyr42Ser (rs115908597) Y42S		Pro24Ala (rs139299015) P24A		Result	Website
	Score	Prediction	Score	Prediction		
M-CAP	*	*	0.005	Benign	Pathogenicity threshold: > 0.025.	http://bejerano.stanford.edu/mcap/
SIFT4G Predictions	0.004	Deleterious	0.339	Tolerated	Score range: 0 to1 (<= 0.05 damaging / > 0.05 tolerated).	https://sift.bii.a-star.edu.sg/www/SIFT_dbSNP.html
PolyPhen-2_HVAR	0.831	Probably damaging	0.000	Benign	Score range: 0 (benign) to1 (damaging). Probably damaging, possibly	http://genetics.bwh.harvard.edu/pph2/index.shtml
PROVEAN	-0.86	Neutral	-0.56	Neutral	Default score threshold: -2.5 (<= -2.5 deleterious / > -2.5 neutral).	http://provean.jcvi.org/
WS-SNPs&GO	0.280	Neutral	0.106	Neutral	Score range: 0 to 1 (Probability score: > 0.5 disease-associated).	http://snps.biofold.org/snps-and-go/
MutPred2	0.625	Possibly pathogenic	0.209	Neutral	Score range: 0 to 1 (General pathogenicity score: ≥ 0.50).	http://mutpred.mutdb.org/#qform
SNAP	2	Effect	-54	Neutral	Score range: -100 to 100 (≥1 effect).	http://www.rostlab.org/services/SNAP
Fathmm	0.52	Tolerated	0.67	Tolerated	Pathogenicity threshold: < 0.	http://fathmm.biocompute.org.uk/inherited.html
Mutation assessor	1.04	Low impact	0.345	Neutral	Score cutoff: 0.8 neutral and low impact / 1.9 low impact and medium impact	http://mutationassessor.org/r3/
PANTHER-PSEP	455	Probably damaging	91	Probably benign	Length of time: > 450my probably damaging / 450my > time > 200my	http://www.pantherdb.org/tools/csnpScore.do
Mutation Taster	0.999999999606647 - A	Disease causing	0.999999999606647 - P	Polymorphism	Prediction: A. Disease causing: probably deleterious / D. disease	http://www.mutationtaster.org/
Revel (GRCH 38)	0.168	Benign	0.081	Benign	Score range: 0 to 1 (>0.50 likely disease causing / <0.50 likely benign).	https://sites.google.com/site/revelgenomics/downloads

* MCAP score is not available for some alleles - Location 1:203317274

Reference
Jagadeesh et al., 2016
Vaser et al., 2016
Adzhubei et al., 2010
Choi and Chan, 2015
Capriotti et al., 2013
Pejaver et al., 2020
Bromberg and Rost, 2007
Shihab et al., 2013
Reva, Antipin and Sander, 2011
Tang and Thomas, 2016
Schwarz et al., 2014
Loannidis et al., 2016
